# Temperature rise during removal of fractured components out of the implant body: an *in vitro* study comparing two ultrasonic devices and five implant types

**DOI:** 10.1186/s40729-015-0008-0

**Published:** 2015-03-20

**Authors:** Eric W Meisberger, Sjoerd J G Bakker, Marco S Cune

**Affiliations:** 1 University Medical Center Groningen, Center for Dentistry and Oral Hygiene, Department of Fixed and Removable Prosthodontics and Biomaterials, The University of Groningen, Gebouw 3216, kamer 206, A. Deusinglaan 1, 9713 AV Groningen, The Netherlands; 2 Department of Oral-Maxillofacial Surgery, Prosthodontics and Special Dental Care, St. Antonius Hospital Nieuwegein, Koekoekslaan 1, 3435 CM Nieuwegein, The Netherlands

**Keywords:** Implant, Abutment, Complication, Fracture

## Abstract

**Background:**

Ultrasonic instrumentation under magnification may facilitate mobilization of screw remnants but may induce heat trauma to surrounding bone. An increase of 5°C is considered detrimental to osseointegration. The objective of this investigation was to examine the rise in temperature of the outer implant body after 30 s of ultrasonic instrumentation to the inner part, in relation to implant type, type of ultrasonic equipment, and the use of coolants *in vitro*.

**Methods:**

Two ultrasonic devices (Satelec Suprasson T Max and Electro Medical Systems (EMS) miniMaster) were used on five different implant types that were provided with a thermo couple (Astra 3.5 mm, bone level Regular CrossFit (RC) 4.1 mm, bone level Narrow CrossFit (NC) 3.3 mm, Straumann tissue level regular body regular neck 3.3 mm, and Straumann tissue level wide body regular neck 4.8 mm), either with or without cooling during 30 s. Temperature rise at this point in time is the primary outcome measure. In addition, the mean maximum rise in temperature (all implants combined) was assessed and statistically compared among devices, implant systems, and cooling mode (independent *t*-tests, ANOVA, and post hoc analysis).

**Results:**

The Satelec device without cooling induces the highest temperature change of up to 13°C, particularly in both bone level implants (*p* < 0.05) but appears safe for approximately 10 s of continuous instrumentation, after which a cooling down period is rational. Cooling is effective for both devices. However, when the Satelec device is used with coolant for a longer period of time, a rise in temperature must be anticipated after cessation of instrumentation, and post-operational cooling is advised.

**Conclusions:**

The in vitro setup used in this experiment implies that care should be taken when translating the observations to clinical recommendations, but it is carefully suggested that the EMS device causes limited rise in temperature, even without coolant.

## Background

Complications in implant dentistry are generally divided into biological and mechanical complications. Mechanical complications include fracture of the implant body or prosthetic components such as chipping of ceramic material as well as loosening or fracture of implant abutments or fixation screws. This has a documented prevalence of 6 to 13% and 0.4 to 2% respectively after 5 years [1-7].

With respect to the etiology of screw loosening, several causes are to be considered [8]. Screw-nut systems generally become unstable when the load that is applied to the system exceeds that of the preload of the screw that causes a clamping force preventing separation of the joint [9]. Preload is proportional to the tightening torque at placement. Hence, tightening torque on the one hand has a significant effect on screw loosening [10]. Embedment relaxation or settling can be overcome by retorquing abutment screws after a certain period of time, increasing joint stability [9,11]. On the other hand, the magnitude of forces applied to the system is of major influence. The transfer of high forces can be generated by bruxers, through non-occlusal loading or because of a non-passive fit of suprastructures [8,12-15]. Other factors that are of influence of a systems’ resistance to screw loosening include lubrication, screw design, screw material, and surface characteristics [16-20]. Especially implant designs with an external hex configuration are prone to abutment screw loosening [21].

Fracture of abutments or abutment screws can be contributed to acute trauma, chronic overload, production flaws, or errors in screw-nut design. If fractured screw components cannot be removed, it may render the implant unrestorable or forces the dentist to creative solutions, such as cementable components. Fortunately, fractured screw components will generally be loose because the preload has not been retained. In that case, they may be removed by manipulating them counter clockwise with a straight probing instrument. On occasion, screw remnants cannot be mobilized, and removal remains a clinical challenge. Careful instrumentation when attempting to remove them should prevent damage to the internal thread of the implant and its surrounding tissues. The use of a fine-tipped hand instrument, a round burr turning counter clockwise, or drilling a slot in the screw in order to get more grip can be attempted to loosen the fragment. Specific instrumentation to remove broken screws from implants is available from most implant suppliers.

The use of ultrasonic equipment under adequate magnification may facilitate removal. It generates heat. Instrumentation without a coolant likely increases the temperature of the implant body and could cause tissue damage, in particular be harmful to osseointegration. The use of a coolant could be effective, but compromises visibility considerably, hence increases the risk of damaging the internal configuration of the implant.

Results from an experimental heat conduction model investigating the ranges of temperature gradients occurring in implants demonstrate that a 60°C heat source causes a heat front exceeding 47°C and advances more than 3 mm down an implant within 1 s [22]. Temperatures over 47°C for more than a minute cause necrosis of cortical bone [23-25]. Some studies investigating the potential harmfulness of intraoral abutment preparation or plaster on implants to osseointegration mention this threshold as harmful [26-28]. It has been postulated that a rise in temperature to 42°C causes denaturation of osteoblasts and should be considered the temperature threshold of transient changes in bone [25]. This threshold was used by others when investigating the potentially damaging effect to the implant-bone interface as a result of drinking hot beverages [29-31]. Also from endodontic literature regarding the removal of metal endodontic posts, concerns have been raised based on observations from *in vitro* experiments with respect to potentially detrimental heat transformation through dentine while ultrasonically manipulating the post [32-37].

The objective of this investigation is to examine the rise in temperature of the outer surface of an implant body after 30 s of ultrasonic instrumentation of its inner part in relation to type of ultrasonic equipment, implant type, and the use of coolants *in vitro*.

## Methods

Two different types of commercially available ultrasonic devices, set at their lowest intensity for endodontic purpose, were used to instrument the internal portion of five different implant types, either with or without cooling. Intermittent anti-clockwise strokes were made, assuring that the tip was constantly in contact with the inner implant wall, as much as possible mimicking the motion that would have been used in clinical practice.

The ultrasonic devices used were the Satelec Suprasson T Max (Acteon Group, Merignac, France) and the EMS miniMaster (EMS, Electro Medical Systems SA, Nyon, Switzerland) with non-diamant, non-cutting tips ET 20, Satelec, and Instrument A (EMS). The device allowed for internal cooling of the tip with the cooling liquid at 31°C during instrumentation.

**Figure 1:**
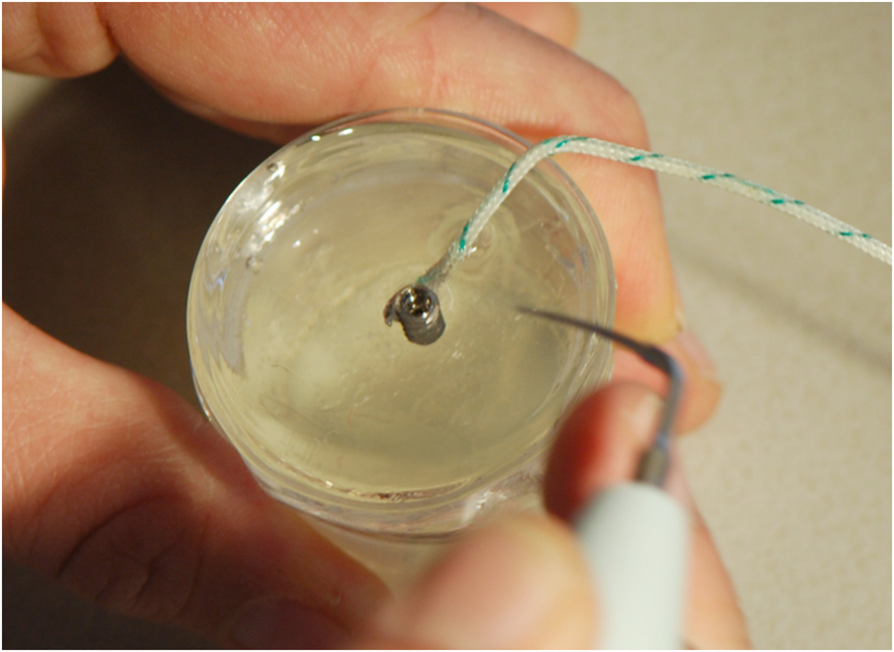
**Implant embedded in epoxy resin with thermocouple at the outer surface.**

The implants used were from different brands; all 8-mm long but with various diameters and designs: Astra 3.5 mm (Dentsply Implants, Mölndal, Sweden), bone level Regular CrossFit (RC) 4.1 mm, bone level Narrow CrossFit (NC) 3.3 mm, Straumann tissue level regular body regular neck 3.3 mm (Straumann, Basel, Switzerland), and Straumann tissue level wide body regular neck 4.8 mm (Straumann AG, Basel, Switzerland). A single implant per group was used. They were embedded in epoxy resin, with a thermocouple (TC-08, Pico Technology, St. Neots, Great Britain) glued to the outer implant surface, at a level corresponding with the anticipated marginal bone level in uncompromised conditions. The change in temperature was registered for 30 s, followed by a 30-s cooling down period, at 5-s intervals (Figure 1).

### Statistical analysis

The primary outcome variable was defined as the difference in temperature between the start of instrumentation and after 30 s when comparing the different implants and the maximum rise in temperature (deltaTmax) where results were averaged per experimental condition (type of device, with or without coolant). All tests were performed three times, and the results were averaged per condition. Differences between several experimental conditions were analyzed by means of independent *t*-tests and univariate analysis of variance, after verification of normal distribution by human eyeballing and the Kolmogorov-Smirnov test. Where appropriate, post hoc analysis was performed using the Student-Newman-Keuls multiple comparison test. The value for *α* was set at 0.05 to distinguish statistical significancy.

## Results

**Figure 2:**
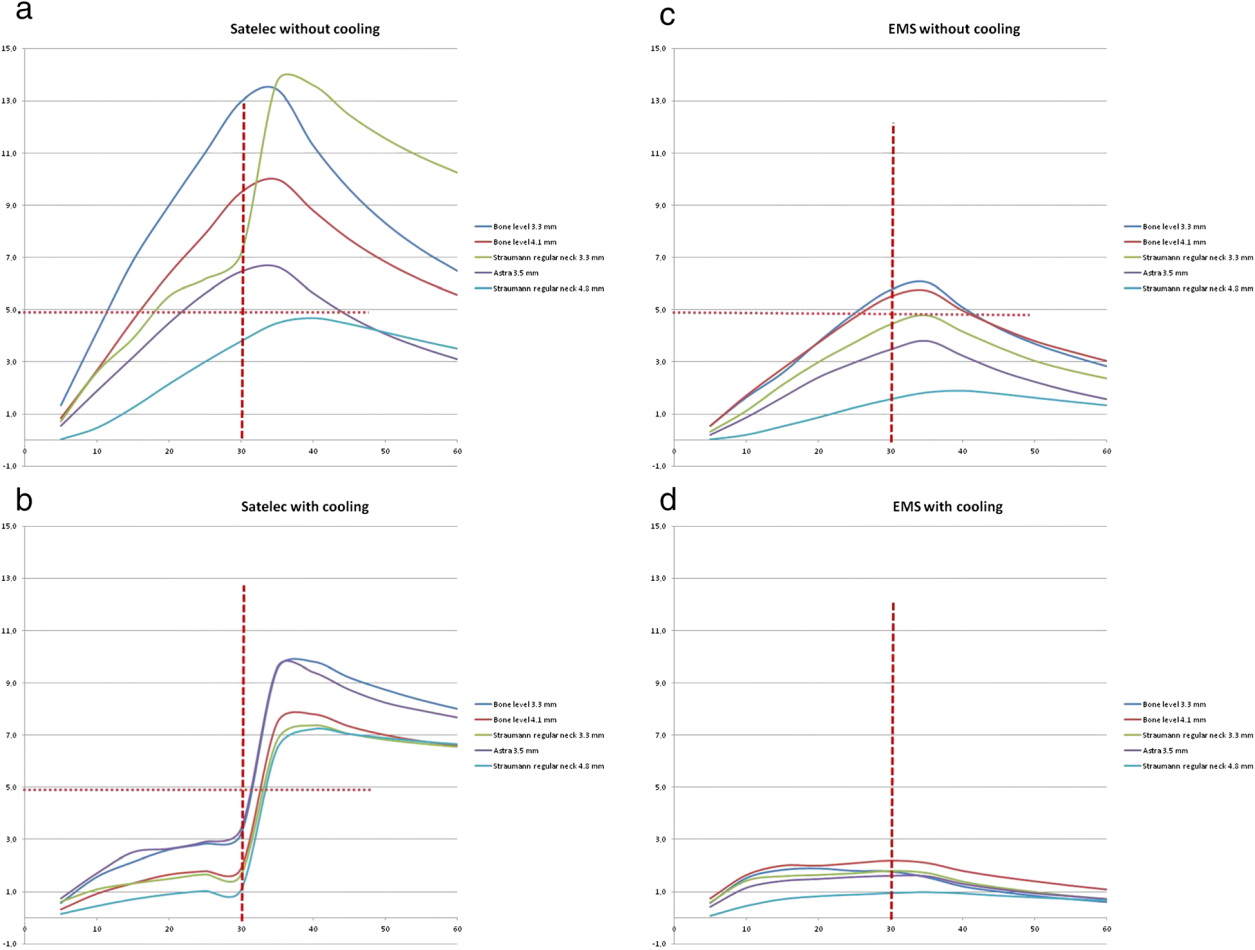
**Results for all implants instrumented with two tested ultrasonic devices, either with or without cooling. (a)** Temperature rise when instrumenting with the Satelec ultrasonic device without cooling. The horizontal dotted line denotes the assumed critical rise in temperature. Temperature rise at 30 s: bone level 3.3 mm > bone level 4.1 mm > Straumann regular neck 3.3 mm = Astra 3.5 mm = Straumann regular neck 4.8 mm. **(b)** Temperature rise when instrumenting with the Satelec ultrasonic device with cooling. The horizontal dotted line denotes the assumed critical rise in temperature. Temperature rise at 30 s: bone level 3.3 mm = Astra 3.5 implant > Straumann regular neck 3.3 mm = Straumann regular neck 4.8 mm. Temperature rise at the bone level 4.1 implant lies in between the bone level 3.3 mm and Astra 3.5 mm implant and both Straumann implants, but not significantly different from either of these implants. **(c)** EMS without cooling. Temperature rise at 30 s: bone level 3.3 mm = bone level 4.1 mm > Straumann regular neck 3.3 mm = Astra 3.5 mm = Straumann regular neck 4.8 mm. **(d)** EMS with cooling. No statistically significant differences between the implant types at 30 s.

The results for all implants instrumented with the two tested ultrasonic devices, either with or without cooling, are presented in Figure 2a,b,c,d.

For the Satelec device, applied *without* coolant, only the temperature of the Straumann wide body regular neck implant never exceeded the 50 threshold. The data proved normally distributed (Kolmogorov-Smirnov test, *p* > 0.05). Analysis of variance indicated statistically significant differences among the implant types (*F* = 33.3, *df* 4, *p* < 0.001). The highest mean temperature increase at 30 s was seen around the 3.3-mm bone level implant (13.0°C), followed by the 4.1-mm bone level implant (9.5°C), and subsequently by the other three implant types (Student-Newman-Keuls (SNK) test, Figure 2a). When coolant was used during instrumentation, an increase of temperature exceeding 5°C was not seen. There were some differences between the implant types (ANOVA, *F* = 6.4, *df* 4, *p* < 0.01), primarily between the bone level 3.3 mm and Astra 3.5-mm implant on the one hand and both Straumann tissue level implants on the other hand (SNK test). Interestingly, when instrumentation and cooling had stopped, the outer temperature of all implants raised markedly above the 5°C critical threshold during the following 10 s, for both bone level implant types up to approximately 10°C (Figure 2b).

For the EMS device when applied *without* cooling, an increase at 30 s above the threshold was only seen for the 3.3- and 4.1-mm bone level implant types (approximately 6°C), which was statistically significantly higher than the rise in temperature seen in the other three implant types (ANOVA, *F* = 3.4, *df* = 4, *p* = 0.04, and SNK test, Figure 2c). Cooling the EMS device proved pretty efficient at 30 s, without differences between the groups. Only a mild increase of the outer temperature was observed to a maximum of 2°C. It takes some time for cooling to take effect. When instrumentation and cooling is stopped, no increase of outer implant temperature occurs, which contrasts the findings with the Satelec device (Figure 2d).

The mean maximum rise in temperature (deltaTmax, all implants averaged per experimental condition) was reached at 30 to 40 s for all conditions. There was a significant difference for the Satelec device without cooling (mean 9.6, SD 1.6°C) and the EMS device without cooling (mean 4.3, SD 2.0°C) (*t*-test, *t*(28) = 4.7, *p* < 0.001). When using coolant, the deltaTmax for the Satelec device (mean 8.3, SD 1.3°C) was significantly higher than for the EMS device (mean 1.6, SD 0.6°C) (*t*-test, *t*(28) = 17.9, *p* < 0.001).

## Discussion

Several techniques have been described to deal with biomechanical complications. Acquiring adequate visibility and access is essential to success which will require the use of a dental microscope.

Several mechanical approaches to remove screw remnants can be employed. Generally, after identifying the position and condition of the screw remnant, it can be carefully removed using manual instrumentation. A fine-tipped hand instrument may be wedged between screw and implant. If this fails to do the trick, the use of a round burr applying occlusal pressure and turning counter clockwise may loosen the fragment. When a flared burr is wedged between implant and screw remnant, the drill should make a clockwise action, to direct the screw in the counterclockwise direction. Cutting a deep slot in the screw and subsequently removing it with a bladed screw driver has also been advised. The use of rotary instrumentation may cause damage to the cranial inner portion of the implant body. If that occurs, even when the fragment (finally) loosens, it will not screw out, and this complicates matters considerably.

Most implant suppliers, and also some third parties, provide specific instrumentation to remove broken screws from implants (i.e., Certain screw removal kit, Biomet 3I, Palm Beach Gardens, United States; screw removal kit NobelReplace, Nobel Biocare, Göteborg, Sweden; Neo screw remover kit, Neobiotech Co, South Korea). In general, they screw into the center of the screw remnant, which facilitates another burr to grip it and remove it counter clockwise or fragmentate the screw remnant.

The use of ultrasonic equipment has also proven to be effective in dislodging fixed screw remnants at the risk of damaging the inner portion or overheating the implant. Hence, the use of magnification is a must and cooling may be advisable, but compromises the vision of the operator. The present study evaluated heat accumulation *in vitro* and the efficiency of cooling when using two types of ultrasonic equipment to the inner portion of several implants.

The degree to which a material is able to transfer heat is called thermal conductivity. It can be defined as the time rate of transfer by conduction, through unit thickness, across unit area for unit temperature gradient. Differences in design and wall thickness of the implants used in the present study account for the variation in outcome (i.e., Straumann tissue level 4.8 implant less effected), considering the fact that all implant types used are made from the same material: grade 4 commercially pure (CP) titanium. The thermal conductivity of CP titanium is relatively low compared to, for instance, that of steel (1/4) and aluminum (1/13). On the other hand, it is approximately 60% higher than that of grade 5 titanium alloy (16.3 W · m^−1^ · K^−1^ for grade 4 CP titanium compared to 7.2 W · m^−1^ · K^−1^ for grade 5 Ti-6Al-4 V). The former material is used in several other implant brands than the ones used here [38], and as a consequence, the results cannot be extrapolated to those systems. The data from this *in vitro* experiment can only be generalized to the clinical situation bearing in mind some inherent limitations and assumptions. The epoxy resin used does not resemble alveolar bone, its structure, water content, and potential to cope with thermal challenges. It is unlikely that wall thickness, design, and material between different implants from the same implant type will vary because of the high degree of current precision and standardization achieved during the fabrication of implants. Consequently, only one specimen per brand was used. To correct for variation during the instrumentation of the ultrasonic device, the experiment was performed three times.

The results show that both 1. the type of implant and 2. the type of ultrasonic device (and in especially the use of coolant) affect the amount of temperature rise to the outer implant surface. Both bone level implants in particular appear to heat up the most. Without the use of coolant, the heat accumulation was much higher with the Satelec compared to the EMS device and exceeded the theoretical threshold for permanent biological damage after 10 to 15 s of continuous instrumentation (Figure 2a,c) and differed statistically significant at deltaTmax. There may be several explanations for this, but most likely, the produced energy at the point of the tip for the Satelec device was higher. Both devices were set at their lowest possible level. The present authors have not been able to verify what energy levels are actually produced. The former is presumed linear to the frequency and the deflection/amplitude of the tip. The frequency is mentioned in the product documentation (Satelec: 27 to 33 kHz and the EMS: 24 to 32 kHz) but the amplitudes differ per tip and are not disclosed. The effect of the difference in coolant spray between the two instruments is also a factor that needs considering when interpreting the data.

Cooling proves effective for both systems, increasing the outer implant temperature by an acceptable 1°C to 3°C during continued instrumentation; however, as already stated, the spray will blur the vision of the operator.

One would expect the temperature to drop immediately after cessation of instrumentation and cooling, but the peak temperature is reached some seconds later for all experimental conditions, so regardless of device, use of coolant, or implant type (Figure 2a,b,c,d). Others, instrumenting endodontic posts in natural teeth [32,33], also saw a ‘lag’ period with a rise in temperature after cessation that lasted up to 9 s post-instrumentation. They left it unexplained. We offer two possible explanations. Firstly, ultrasonic instrumentation causes the implant wall and its surrounding tissues (in this case, resin and thermocouple, but *in vivo*, this would be surrounding alveolar bone) to vibrate and generate heat. Vibration continues some time after cessation of instrumentation, explaining the continued rise in temperature. Secondly, after cooling has stopped, generated and stored heat in the surrounding ‘tissues’ may flow back to the outer wall of the implant that is no longer cooled from the inside and raises the temperature. The potentially damaging rise in temperature (‘explosion’) seen for the Satelec device, but not for the EMS device when instrumentation and cooling was stopped, could be explained by these phenomena on the one hand and by the fact that energy produced by the Satelec device was presumably higher on the other hand (Figure 2b).

## Conclusions

It is concluded from this *in vitro* study that heat accumulation and transfer is dependent on the type of ultrasonic device, the use of coolant, and the implant type. The highest rise in temperature is seen when using the Satelec device without coolant on the smaller diameter implants. The EMS device causes limited rise in temperature when used without coolant for less than 10 s, but presumably delivers less energy to the tip, and consequently may be not as effective. A cooling down period is sensible. When used with coolant for a longer period of time, the clinician should anticipate a considerable rise in temperature after cessation of instrumentation, and post-operational cooling is advised.
